# Biorepositories For Global Rare Disease Research: A Narrative Review

**DOI:** 10.1007/s11926-025-01189-6

**Published:** 2025-05-21

**Authors:** Maria Rosa Pellico, Jessica Day, Meera Shah, Belina Y. Yi, Lesley Ann Saketkoo, Christian Lood, Latika Gupta

**Affiliations:** 1 Department of Clinical Sciences and Community Health, University of Milan, Milan, Italy; 2 Division of Rheumatology, ASST Gaetani Pini-CTO, Milan, Italy; 3 Department of Rheumatology, Royal Melbourne Hospital, Parkville, Australia; 4 Inflammation Division, Walter and Eliza Hall Institute of Medical Research, Parkville, Australia; 5 Department of Medical Biology, The University of Melbourne, Parkville, Australia; 6 Department of Rheumatology, Indraprastha Apollo Hospital, New Delhi, India; 7 Division of Pediatric Allergy, Immunology, and Rheumatology, Johns Hopkins University, Baltimore, MD USA; 8 New Orleans Scleroderma and Sarcoidosis Patient Care and Research Center, New Orleans, LA USA; 9 Myositis International Health & Research Collaborative Alliance (MIHRA), Baltimore, USA; 10 LSU and Tulane University Schools of Medicine, New Orleans, LA USA; 11 Division of Rheumatology, University of Washington, Seattle, WA USA; 12 Department of Rheumatology, Royal Wolverhampton Hospitals NHS Trust, Wolverhampton, UK; 13 School of Infection, Inflammation and Immunology, College of Medicine and Health, University of Birmingham, Birmingham, UK; 14 Division of Musculoskeletal and Dermatological Sciences, School of Biological Sciences, Centre for Musculoskeletal Research, The University of Manchester, Manchester, UK; 15 Francis Crick Institute, London, UK

**Keywords:** Biobank, Biospecimen, Rare disease, Connective tissue disease, Myopathies, Myositis

## Abstract

**Purpose of this Review:**

Rare diseases, although individually infrequent, collectively impact a substantial number of people. Collaborative translational research using biospecimens is essential for advancing our understanding of the diverse characteristics and pathophysiology of rare diseases. Biobanks play a pivotal role in this endeavor by collecting, processing, transporting, and storing biospecimens, thereby serving as invaluable resources for medical research. In this review, we explore currently available biobanks, with a specific focus on those dedicated to rare rheumatic diseases. We also examine accessible best practice guidelines for establishing and maintaining high-quality biobanks, discuss the limitations and propose future directions for enhancing biobanking efforts in rare disease research.

**Recent Findings:**

Advances in molecular and genomic technologies have expanded the role of biobanks, enhancing biomarker discovery and precision medicine. However, despite growth in biobanking capabilities, key challenges persist concerning ethics, interoperability, and biospecimen exchange, prompting active responses by various regulatory and governing bodies.

**Summary:**

Biobanking has transformed rare disease research. Strengthening national and international collaborations is essential for driving progress in this field and accelerating the development of novel therapeutic and precision medicine approaches.

## Introduction

Rare diseases (RD) are defined as those affecting fewer than 1 in 2000 individuals in the community as per The European Commission or fewer than 200,000 people in the United States according to the National Organization for Rare Disorders (NORD) [1]. Several systemic connective tissue diseases in rheumatology, including idiopathic inflammatory myopathies (IIM), systemic lupus erythematosus (SLE), and systemic sclerosis meet these criteria [2]. Investigating these conditions is particularly challenging due to their low prevalence and clinical heterogeneity.

A major obstacle in studying rare diseases is assembling sufficiently large and diverse patient cohorts, given the scarcity of specialized research centers. While meaningful scientific discoveries have been made within single institutions, multi-center collaboration enhances the robustness and generalizability of findings. One of the most significant hurdles in translational research on rare diseases is limited access to infrastructure supporting biospecimen collection, storage and analysis.

Biobanks play a crucial role in overcoming these challenges. By providing centralized, facilitating access across sites, biobanks enable broader collaboration and improve statistical power. This broader access can lead to a more comprehensive understanding of rare systemic rheumatic diseases which increases the likelihood of identifying novel biomarkers that are accurate and representative of the wider patient populations, aiding in early diagnosis and treatment [3].

However, despite their importance, biobanks dedicated to rare systemic rheumatic diseases remain limited, with most concentrated in developed countries and major urban centers. This geographical clustering restricts the diversity of patient populations, potentially limiting the representativeness of disease characteristics in global research. Moreover, there is a lack of evidence-based, standardized, and widely accessible protocols for handling biospecimens in rare systemic rheumatic disease research, further complicating progress in the field. These challenges are particularly pronounced for early-career researchers, especially those at smaller and emerging and lower -income countries, who may face substantial barriers in accessing the infrastructure and resources needed for biobanking and biospecimen research.

In this narrative review, we examine the current landscape of collaborative biobanks dedicated to rare systemic rheumatic diseases, addressing unmet needs, international standards and unique challenges in the field.

## Methods

This review employed a structured, narrative approach to the literature, incorporating elements of systematic review methodology without adhering to the formal protocols.

### Literature Search Strategy

A comprehensive literature search was conducted to identify relevant studies and publications on biobanks in rare rheumatic diseases, covering articles from January 2011 to April 2024. The search strategy was designed to capture a wide range of research articles, reviews, guidelines, and reports, with a focus on the establishment, operation, and challenges of biobanks, particularly in relation to rare rheumatic diseases. The search was performed across multiple electronic databases, including PubMed, Scopus, Web of Science, and Google Scholar.

The search terms used included combinations of the following keywords: “biobank”, “biorepository”, “biospecimen”, “rheumatic diseases”, “rare diseases”, “rheumatology”, “standard operating procedures”, “ethics”, “multi-center”, “collection methods”, “sample storage”, “data sharing”, and “reproducibility”. Boolean operators (AND, OR) were employed to refine the search results [4].

### Inclusion and Exclusion Criteria

Articles were included if they focused on biobanks related to rheumatic diseases, with particular emphasis on rare or orphan diseases within the rheumatology field. Studies discussing the establishment, management, or challenges of biobanks, including ethical, operational, and scientific aspects, were considered relevant. Publications providing information on biospecimen collection, processing, storage, and data sharing practices, especially those highlighting multi-center biobank studies, were also included.

Exclusion criteria included articles that did not specifically address biobanks in the context of rheumatic diseases, studies that focused on common rheumatic diseases without addressing issues unique to rare diseases and publications not available in full-text or those published in languages other than English.

#### Data Extraction and Synthesis

The identified articles were screened by title and abstract to assess their relevance to the objectives of this review. Full-text versions of potentially relevant studies were retrieved and reviewed in detail. Data extracted included the type of biobank, sample types, geographic and institutional coverage, ethical considerations, and challenges related to the biobanking of rare rheumatic diseases. Emphasis was placed on identifying common themes, challenges, and best practices that could inform the development of biobanks in resource-limited settings.

The results of the selected studies were synthesized narratively, highlighting key findings, gaps in knowledge, and areas for future research. A thematic approach was used to organize the data, focusing on the ethical, operational, and scientific challenges faced by biobanks in the context of rare rheumatic diseases. Additionally, the review examined examples of successful biobanks and their contributions to advancing research in this field.

## Defining Biobanks and Their Role

Biobanks collect, store, and process biospecimens to facilitate research on diverse diseases. These repositories integrate biological samples with clinical data, enabling large-scale studies and providing insights into population health and disease mechanisms. Biobanks can be broadly categorized into population-based and disease-oriented resources, each serving distinct research needs [5].

Population-based biobanks, such as the UK Biobank, tend to operate on a large scale, collecting biological samples from volunteers, often without specific inclusion or exclusion criteria. These biobanks enable large-scale genetic studies and development of risk assessment models [6]. Another notable example is the Swedish Malmo Diet Cancer Study, which has followed 30,000 participants for over 30 years, enabling important discoveries regarding risk factors for a variety of diseases including cancer, cardiovascular disease and rheumatic conditions like giant cell arteritis and rheumatoid arthritis [7]. Similarly, the China Kadoorie Biobank, with over 500,000 participants, provides regionally anchored, longitudinal data that has supported investigations into autoimmune disease risk factors in East Asian population [8]. The primary aim of population-based biobanks is to investigate the interplay between genetic susceptibility and environmental exposures in disease development, combining biospecimen research with comprehensive clinical and demographic data.

Disease-oriented biobanks, in contrast, focus on specific conditions and enable investigations into pathogenesis, biomarker discovery, diagnostics, long-term patient monitoring and identification of potential therapeutic strategies. These biobanks provide high-quality biospecimens linked to detailed clinical information. By integrating a wide array of data from numerous biological samples, research groups can develop large-scale research projects and gather information on the studied population. A key example is the Systemic Lupus Erythematosus International Collaborating Clinics (SLICC), an international repository collecting DNA, plasma, and serum samples on SLE [9].

## Operational Challenges in Biobanking

While biobanks are essential for medical research, their operation is often hindered by infrastructure limitations, governance issues, and lack of standardization. Effective and efficient biobanking requires rigorous biospecimen collection, processing, storage, distribution and clinical annotation, but inconsistencies in protocols across institutions can compromise sample quality and data integrity. Additionally, many biobanks operate in isolation, leading to fragmentation, reduplication of efforts and limited data sharing.

To better understand and address these challenges, Shickle et al. classified biobank networks into six operational categories—‘storage’, ‘bring-and-share’, ‘catalogue’, ‘partnership’, ‘contribution’, and ‘expertise’—based on their operational and governance structures rather than their research focus. For example, storage networks primarily offer centralized infrastructure, developed to provide cost-effective shared storage solutions, while expertise networks focus on knowledge-sharing and standardization rather than sample exchange. This non-mutually exclusive, governance- and operational-focused classification provides a framework for improving biobanking networking and identifying solutions from an operational perspective [10, 11].

Cross-departmental collaboration, such as partnerships with pathology or clinical biochemistry laboratories, has been shown to improve sample collection accuracy and overall biobank quality, as exemplified by the Danish Rheumatologic Biobanks [12]. However, variability in sample processing and the absence of harmonized protocols across different biobanks remain major concerns. Differences in collection, handling and storage can introduce confounding factors, potentially compromising the validity of findings due to uncontrolled preanalytical variables. Additionally, accurate and standardized clinical data is also vital for linking molecular profiles to disease outcomes [13, 14].

To mitigate these challenges, international efforts have focused on establishing standards for sample collection, storage, and distribution. The International Society for Biological and Environmental Repositories (ISBER) provides comprehensive guidance through its Best Practices for Repositories, emphasizing risk and quality management, ethical and social considerations, and uniform specimen handling [15].

Similarly, the International Standards Organization (ISO) has developed ISO 20387:2018, an international standard specifically for biobanking. This certification sets globally recognized quality and competency requirements for biorepositories, covering sample collection, preparation, data management, storage, and distribution [16]. Institutions adhering to this standard demonstrate high-quality biobanking practices, fostering international collaboration and improving research reproducibility. These efforts by ISBER and ISO have been instrumental in synchronizing biobanking practices globally.

The field of oncology also provides a successful model for standardization, with The National Cancer Institute Best Practices for Biospecimen Resources serving as a benchmark for consistent biospecimen handling [17].

## Establishing and Maintaining Disease-Specific Biobanks in Rheumatology: Logistical Considerations

### Biospecimen Collection and Handling

**Fig. 1:**
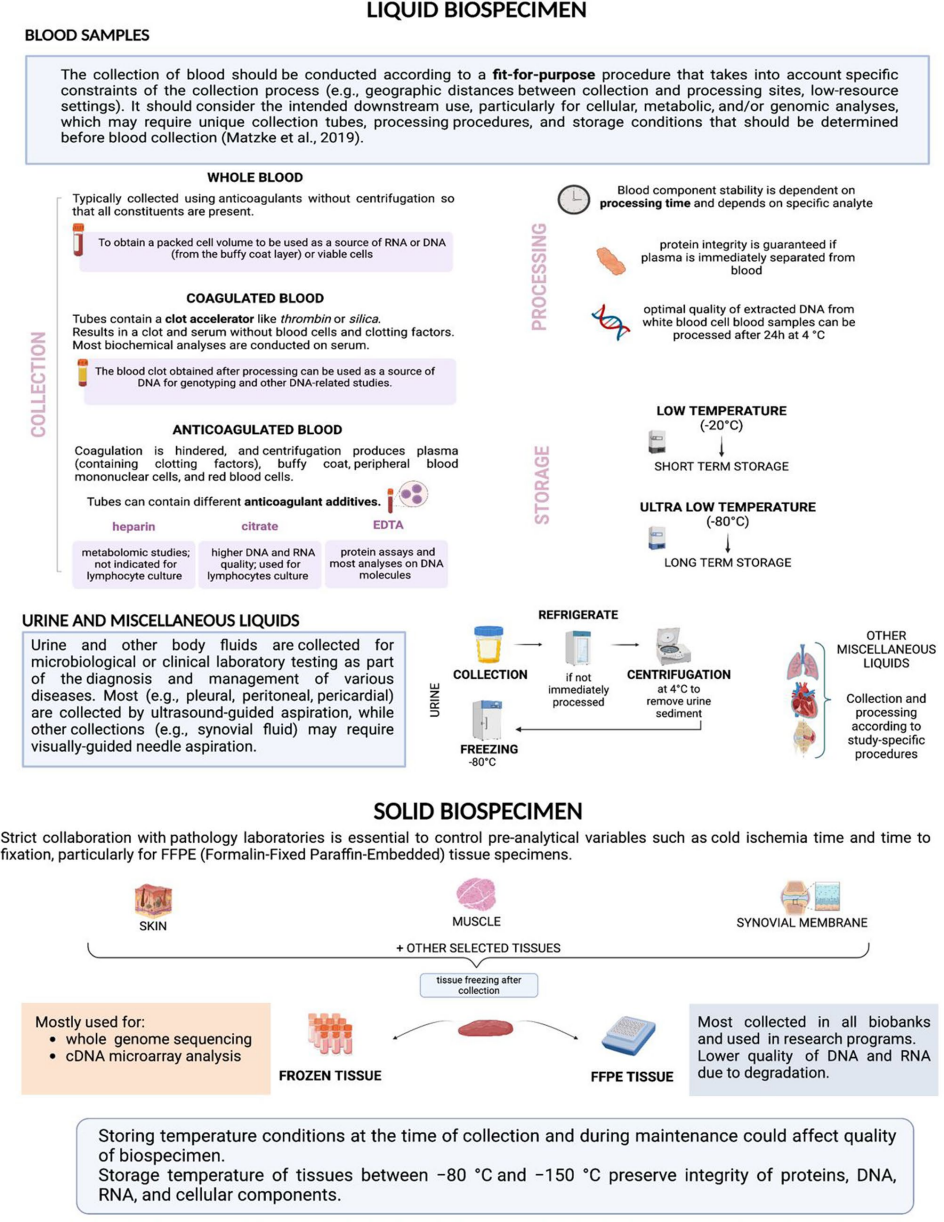
Overview of Liquid and Solid Biospecimen Collection, Processing, and Storage

The collection and storage of biospecimens for biobanking has historically lacked standardized practices, particularly in the pre-analytical steps [18]. Human biological samples encompass a wide variety of materials, such as tissues, organs, DNA or RNA extracts, blood, bodily fluids, cell lines, cell suspensions, and plasma. Figure 1 shows the steps involved in liquid and solid biospecimen collection, processing, and storage. The standardization of operating procedures directly influences the quality and usability of samples in downstream research. Standard operating procedures for sample procurement, processing, and storage should adhere to the best practices recommended by the ISBER [19]. Adherence to these procedures ensures sample integrity for downstream research applications.

### Pre-Analytical Errors and Biosample Bias

Pre-analytical variables refer to any and all procedures that occur during sample collection, prior to sample analysis. The pre-analytics phase consists of two phases namely the pre-acquisition phase (the sample is not under the supervision and control of biobank personnel) and the acquisition phase (the sample comes under the supervision and control of biobank personnel), and are major sources of errors in laboratory diagnostics [18]. This includes patient identification, physical sample collection, sample transportation to the testing site and sample preparation. Variability in pre-analytic factors has significant impact on the reproducibility of results, especially in sensitive analyses such as metabolomics, proteomics, and biomarker research for rare rheumatic diseases [20].

Pre-analytical factors crucial for sample integrity include:

1. Tube type: Additives impact analyte composition
2. Clotting time: Affects coagulation and biomolecule release
3. Transport/incubation time: Influences enzymatic activity
4. Transport temperature: Affects protein and metabolite stability
5. Storage conditions: Impact long-term sample preservation
6. Processing time: Critical for metabolite profile accuracy

Biosamples should be labeled pseudonymously and stored in aliquots. For long-term aliquoting, it is recommended to store samples from an individual in multiple 0.5- or 1.0-mL aliquots. It is essential to minimize resampling or freeze–thaw events, both of which have been shown to impact downstream analysis [20, 21]. Examples of key pre-analytical factors and their impact on laboratory diagnostics across various assays and sample types are provided in Table 1.

**Table 1:** Pre-analytical factors and their impact on laboratory diagnostics across different assays and sample types

Author Year	Sample Type	Assay Type	n	Pre-analytic factor	Impact	Processing Part Without Impact
Kirk et al. 2008 [22]	Urine	ELISA	13	Time at room temperature, protease inhibitors, pH alteration	VEGF levels were significantly impacted by time at room temperature, with higher values observed between 3–7 h. Protease inhibitors and pH alteration had no significant impact	No significant impact of protease inhibitors or pH alteration on VEGF levels
Timms et al. 2007 [23]	Serum	SELDI-TOF MS	25	Clotting time, transport time, storage	Extended transport/incubation at room temperature led to proteolysis, altering protein profiles significantly	Clotting time had minimal impact when samples were on ice
Webster, MJ. 2006 [24]	Postmortem Brain	RNA and Protein Analysis	450	pH, postmortem interval, agonal state	pH was a better predictor of RNA integrity (RIN) than postmortem interval. Certain diagnostic groups had significantly lower pH values without corresponding lower RIN values, indicating potential metabolic abnormalities	RNA integrity was not significantly affected by PMI within certain ranges
Banks et al 2005 [25]	Plasma and Serum	SELDI-TOF MS	10	Time delay before processing, tube type, anticoagulant	Time delays before processing significantly altered protein profiles, especially in serum samples. Differences between anticoagulants and tube types also affected results, with specific peaks influenced by platelet activation and clotting	Minimal impact when using citrate as an anticoagulant in plasma samples
Drake et al. 2004 [26]	Serum	MALDI-TOF MS	N/A	Blood collection tube type, tube additives	Different types of blood collection tubes released polymeric components detectable by MALDI-TOF MS, potentially interfering with the analysis of low-molecular-weight serum polypeptides	Plasma tubes showed fewer interfering components compared to serum tubes
Breit et al 2004 [27]	Bone Marrow	Microarray, RT-PCR	N/A	RNA extraction protocols, time delays	Time delays between bone marrow aspiration and RNA extraction had significant effects on mRNA gene expression profiles, with 18.8% of probe sets showing deregulation	Immediate processing or stabilization preserved mRNA integrity
Srinivasan et al 2002 [28]	Human Tissues	DNA Microarray, Proteomics	N/A	Fixation and tissue processing	Different fixation methods and tissue processing steps significantly impacted the content and integrity of nucleic acids, affecting downstream molecular analyses	Proper fixation and minimal processing time preserved nucleic acid integrity

### The Challenge and Importance of Complex Biospecimens

Another important concept in biobanking is the management of complex biospecimens. These samples are characterized by features like linkage to other samples from the same donor across different locations or time points, offering invaluable insights into longitudinal changes in disease and gene-environment interactions. However, they are challenging to acquire and manage, particularly when combining smaller cohorts. These biospecimens are essential in health research, especially within biobanks established for longitudinal research studies [29]. The integrated registry-biobank approach offers a promising solution, enabling systematic tracking and management of complex biospecimens and associated longitudinal data across different stages and locations [30].

### Sample Data Recording:

Accurate and standardised recording of sample-related data is critical for biobank functionality. The ISBER advocates for the implementation of harmonized data elements such as specimen type, fixation/stabilization methods, study type and mass/volume, in addition to various other essential annotations. Such standardization enhances biospecimen traceability and database functionality. Key initiatives such as BRISQ (Biospecimen Reporting for Improved Study Quality), SPREC (Standard PREanalytical Code) and MIABIS (Minimum Information About BIobank Data Sharing) have been devised to ensure standardization of data [31–33].

### Clinical Data Acquisition

The acquisition of clinical data is a cornerstone of effective biobanking, necessitating meticulous documentation of participant identifiers, demographic parameters, medical histories, family history, diagnoses, therapeutic interventions, and diagnostic outcomes [34]. Standardization of data collection methodologies is imperative to facilitate interoperability and cross-platform data exchange in research endeavours. Comprehensive documentation should encompass an accessible data dictionary or codebook, delineating data element nomenclature, definitions, data types, permissible ranges, formatting specifications, and validation protocols.

Standalone registries excel in collecting detailed clinical and epidemiological data, tracking disease progression and elucidating natural disease history, but have a limited capacity to support basic and translational research. Rare disease biobanks, on the other hand, provide the infrastructure for basic research, omics research, but typically lack comprehensive epidemiological and clinical data [30].

The shortcomings of each individual modality can be overcome by the integration of registries with biobanks. This synergy facilitates biomarker identification, gene discovery, omics-level investigations, by with linking clinical data and biological specimens [30]. Importantly, integrated registry-biobank models emerge as cost-effective, synergistic solutions, enhancing both data comprehensiveness and research capabilities, facilitating the translation of basic science into clinical applications [35]. Collaborative multi-stakeholder efforts strengthen treatment protocols, data completeness, and industry engagement, ultimately advancing research in rare diseases.

Additionally, when linking large datasets to biobanks, adherence to the RECORD (REporting of studies Conducted using Observational Routinely-collected health Data) statement will ensure consistency in data collection practices. This may strengthen the reporting and reliability of translational observational research [36].

## Informed Consent

Informed consent in biobanking is a complex and evolving issue, balancing the need for comprehensive ethical oversight with practical research needs. A point of contention is the use of broad versus specific consent – specifically whether the provision of broad consent (allowing future, unspecified research) is ethical [37–39].

Proponents of broad consent argue that, when coupled with secure data handling, the right to withdraw participation, and stringent ethical oversight, it respects donor autonomy while enabling critical research [37, 40–42]. Critics, however, contend that broad consent undermines true informed consent since donors cannot anticipate all future uses of their biospecimens [40].

Hartanti et al. developed and piloted a broad informed consent model in Indonesia consisting of an information sheet, comprehension test, agreement sheet and exit survey. When implemented on patients, they found it to be ethically sound, understandable, and acceptable, with recommendations for improving readability and staff coordination [41]. The COVID-19 pandemic further transformed consent practices, emphasizing flexibility and recalibrating research efficacy and participant autonomy [43]. These developments highlighted the need to balance autonomy with societal benefits and adapt ethical protocols for large-scale studies.

Another form of consent that can be implemented is the dynamic informed consent, which claims to be fully informed and involves continuous communication between the researcher and participants. This ensures participants’ decision making, autonomy and real-time consent management, conferring to ethical standards [44].

## Recognition of Contribution

Ensuring proper recognition of biobanking resources is essential for incentivizing data and sample sharing. However, heterogeneous practices and lack of guidelines on how bioresources should be acknowledged in scientific publications have led to inconsistencies in reporting. To address this, the Bioresource Research Impact Factor/Framework (BRIF) was introduced over a decade ago as an initiative to standardize the recognition of bioresources in research, and is currently under further development [45]. The CoBRA (Citing Bioresources in Research Articles) guideline was developed to standardize the citation of bioresources in scientific articles, recommending that each bioresource used in a study be cited in the methods section and listed as an individual reference [46]. Tools like CoBRA and The Open Journal of Bioresources enhance the traceability and visibility of bioresource use, encouraging transparency, recognition, and sharing within the research community [47].

## Interoperability and Biospecimen Exchange

The premise of the Open Science movement is that sharing data, methods and knowledge accelerates progress, a principle especially valued in the rare disease communities [48]. The widely adopted FAIR principles (findable, accessible, interoperable, and reusable), initially established for data sharing in health research, should also be systematically applied to biospecimens. However, the limited exchange leads to inefficient use of research funds, reduced productivity, and hinders reproducibility efforts [49].

To address these limitations, novel data governance models have emerged to manage and share biobank data in more collaborative and responsible ways. These models seek to balance data accessibility, consistent with FAIR principles and open science, with privacy and ethical safeguards, often by distributing governance across networks rather than a single repository [50–52]. One notable example is the use of federated data networks, in which data remain at multiple distributed sites but can be queried or analyzed across those sites under a common governance framework. This approach enables researchers to gain insights from combined data without centralizing sensitive information. For example, the National Patient-Centered Clinical Reasearch Netwotk (PCORnet) in the United States and the international Observational Health Data Sciences and Informatics (OHDSI) network illustrate federated governance: each participating institution keeps custody of its patient data in a standardized format, and analyses are run locally to aggregate results [53, 54].

Implementing FAIR-compliant data stewardship is challenging, necessitating expertise in domain knowledge, information technology systems, data access policies, machine-readable formats, and communication software. To address these barriers, the rare disease community in Europe has embraced the"Bring Your Own Data"(BYOD) workshop concept to help rare disease researchers learn how to make their data FAIR-compliant. Additionally, initiatives like"RDs GO FAIR"and activities of ELIXIR (the European Infrastructure for life science data), BBMRI (Better Biology Makes Reality Interesting), NIH (National Institute of Health), and EURORDIS (European Organization for Rare Diseases) amongst others are working to establish a FAIR-compliant ecosystem that enables new data analysis possibilities in an Open Science environment [48, 55].

Another significant obstacle for researchers involves locating relevant resources via cataloging services like the European Research Infrastructure on Biobanking and Biomolecular Resources (BBMRI-ERIC) Directory. Such directories, often lack sufficient metadata for researchers to accurately assess resource suitability. The BBMRI-ERIC developed the Negotiator tool, which facilitates direct negotiation of access request amongover 600 participating biobanks, streamlining requests across multiple resources while enabling biobanks to manage access decisions independently [56].

## Ethical, legal and societal considerations in biobanking

The operation and collaboration of biobanks are often complicated by ethical, legal and societal challenges, which can restrict biospecimen and data sharing across jurisdictions, hinder international research efforts and impact public trust. Ensuring ethical governance and compliance with legal frameworks is therefore essential to maintain public confidence in biobanking initiatives.

One of the primary ethical concerns is the safeguarding donor privacy and rights. Transparent policies, well-defined governance structures and ethical oversight mechanisms, such as data-access committees and continuous engagement with donors and policymakers are critical for mitigating risks of misuse [57]. Building and maintaining public trust is another major challenge, especially in the era of big data analysis and artificial intelligence. Indeed, a roundtable discussion from the 2021 ISBER annual meeting highlighted the need to improve public trust in biobanking. They found that the awareness regarding biobanking, transparency and communication, as well as community involvement in biobank was particularly limited in Asian countries compared to Australia and United States [58]. Addressing these concerns through education, engagement and transparent policy practices is essential to ensure sustainable participation in biobanking efforts.

Cross-country collaboration presents additional complexities due to differing ethical and legal frameworks, making it difficult to share biological samples and data across jurisdictions. Key barriers include inconsistent consent requirements, differing data protection laws and the absence of globally standardized protocols. The Organization for Economic Co-operation and Development (OECD), an intergovernmental organization with 38 member countries, plays a vital role in shaping the global biobanking landscape by provided guidance on ethical governance, regulatory frameworks and international collaboration [59]. The OECD Guidelines on Human Biobanks and Genetic Research Databases serves as a reference for ensuring transparency, protecting donor rights and fostering trust in collaborative biobank-based research.

Innovative data governance models, such as federated data networks, offer promising solutions for ethical and secure collaboration by allowing data to remain within local institutions while permitting controlled, query-based access across networks. This approach minimizes the need for physical data transfers, thereby reducing risks to privacy and respecting local legal frameworks [50]. However, it also raises ethical considerations around transparency, consent for secondary use, and accountability in distributed systems.

## Sustainability in Biobanking

The rapid increase in biospecimen generation has placed growing pressure on biobanks, underscoring the importance of long-term sustainability. Ensuring the viability of biobanks requires financial stability, cost effective operations, patient and public engagement, standardized protocols, and interoperability. Interoperability means biospecimens and their relevant data can be shared between different biorepositories institutes or databases [60]. Regular evaluation of bioresources (specialized research infrastructures that collect, store, and manage biological specimens and their associated data) also plays a significant role in biobanking sustainability. Beyond tracking the number of biospecimens collected, these evaluations assess how effectively the resource has translated to scientific outputs and outcomes [61].

## Examples of Biobanks in Rheumatology – Challenges and Achievements

Collaborative research is vital in advancing diagnostics and treatments in rare rheumatic diseases. Several international initiatives have established specialized biobanks, such as the SLICC biobank for SLE, the European Scleroderma Trials and Research Group (EUSTAR) biobank for systemic sclerosis, and the MyoCite biobank for IIM. These initiatives underscore the importance of high-quality biospecimens and standardized data in enhancing medical knowledge and patient outcomes for rare diseases [62–64]. Recent initiatives such as the RA-originated Gut Microbial Biobank (RAGMB) highlight the expanding frontier of biobanking by integrating culture-based microbiome collections, advancing our understanding of microbial contributions to autoimmune pathogenesis [65]. The EUSTAR and SLICC biobanks have highlighted the importance of large-scale data in boosting the statistical power of studies, leading to significant discoveries in disease mechanisms and treatment responses [62, 63]. Latin America’s coordinated biologics registries—such as BIOBADAMEX and BIOBADABRASIL—similarly demonstrate how longitudinal data systems can enhance outcome surveillance and inform equitable biobanking strategies in resource-constrained settings [66]. The Childhood Arthritis and Rheumatology Research Alliance (CARRA) has established a biobank specifically for pediatric rheumatic diseases [67]. Impactful biobanks dedicated to rheumatic diseases are presented in Table 2.

**Table 2:** Established biobanks for rare rheumatic diseases

Disease	Biobank/Registries	Bio-specimens	Region	Characteristics
SLE	The Systemic Lupus Erythematosus International Collaborating Clinics (SLICC) [9, 79]	DNA, plasma, serum	North America and Europe, although includes a few centers from Asia and Oceania	• Includes 1,835 + SLE patients, with 1,378 DNA and 9,600 + serum/plasma samples; over 500 patients followed for 10 + years • Supports research on metabolic syndrome, vitamin D deficiency, and cardiovascular risk in lupus • Enables studies on genetic susceptibility and clinical-serologic correlations in SLE • Registry contributed to SLE classification criteria, damage index, disease flare index
	Australian Lupus Registry and Biobank [80, 81]	Whole blood, serum, plasma and urine	Australia, although also developed Asia–Pacific Lupus collaboration	• National registry with 800 + lupus patients across 13 hospitals • Supported 50 + research projects and 8 multi-site studies • Key findings: - Ethnic differences in disease severity - Vitamin D deficiency linked to worse lupus - Steroid use associated with organ damage - Validated LLDAS as a disease control target • Facilitated development of steroid-sparing therapies • Open for collaborative research access
	Lupus BioBank des OberRheins (Lupus BioBank of the upper Rhein “LBBR”) [82, 83]	Unknown	German and French cohort	• Established a globally unique cohort of SLE patients, enhancing research into rare autoimmune diseases.​ • Unified 17 institutions across France, Germany, and Switzerland, fostering transnational cooperation in autoimmune disease research.​
Systemic Sclerosis	The EULAR Scleroderma Trials and Research Group (EUSTAR) [62, 84]	Whole blood, serum, tissues	Europe	• Extensive Patient Registry: Has developed one of the largest international SSc registries, encompassing over 25,000 patients across more than 150 centers worldwide. This comprehensive database facilitates large-scale studies on disease progression, treatment outcomes, and patient demographics.​ • Provides detailed instructions on biospecimen collection
	Collaborative National Quality and Efficacy Registry (CONQUER) [85, 86]	DNA, serum, plasma, whole blood, RNA	U.S	• Launched in 2018 as the first nationwide longitudinal scleroderma registry in the U.S • Builds a collaborative network of U.S. centers to ensure diverse and high-quality data • Enrolled 600 + patients from multiple scleroderma centers by 2021. Defined registry design and utility for improving clinical care and research in systemic sclerosis • Linked early GI involvement with higher healthcare use, highlighting the clinical impact of gastrointestinal symptoms in early disease • Backed by industry partners, including Boehringer Ingelheim and Actelion
	Scleroderma Biobank, Canada (Canadian Scleroderma research group) [87, 88]	Serum, DNA, tissue	Canada	• 1,750 + patients followed for up to 13 years • Over 1,500 variables, emphasizing early disease • Organ-Specific Studies: Includes lung, GI, skin, renal, and cardiac involvement • Lung Cancer Link: Interstitial lung disease associated with increased lung cancer risk • Environmental Risk: Potential link between organic solvent exposure and systemic sclerosis
IIM	MyoCite biorepository [64]	Blood (serum, DNA, plasma), and urine	India	• Collected data on 350 + patients (adult and juvenile) with longitudinal data and bio samples • Collected clinical outcomes using predefined glossary and core set four standardized outcomes • Published protocol of biosample adapted for resource constrained setting • Published findings on diverse aspects: autoantibodies, biomarkers, myositis complications, relapse patterns, and prescription practices- insights specific to the Indian demographic • Described metabolomic profiles across multiple specimens (serum, urine, muscle)
	Swedish Myositis Network (SweMyonet) [89]	Muscle biopsy, serum and DNA	Sweden	• Multicentre, prospective • Collects detailed clinical data on IIM patients, facilitating large-scale studies and improving understanding of disease patterns and outcomes.​ • Research Impact: Identified key predictors of treatment response, such as early therapy and autoantibody status • Integrated Biobanking: Collects blood and tissue samples alongside clinical data, supporting biomarker discovery and personalized medicine efforts
	Pan American League of Associations for Rheumatology (PANLAR) Myositis Registry [90, 91]	Serum, Muscle biopsy	America (both North and South America countries)	• Multicenter, retrospective • Pediatric and adult cases are included
	The Juvenile Dermatomyositis National (UK and Ireland) Cohort Biomarker Study and Repository for Idiopathic Inflammatory Myopathies [92]	PBMCs, serum, genomic DNA and biopsy material	United Kingdom and Ireland	• Established in 2000, it's one of the largest European pediatric IIM registries, with over 285 children enrolled • Collects longitudinal clinical data and biospecimens to support research and improve care • Enabled identification of biomarkers for disease activity and prognosis • Contributed to standardized assessment tools and treatment protocols
Rheumatic diseases	United Kingdom Biobank [93–95]	Blood, urine, saliva	United Kingdom	• One of the world’s largest health databases with data from 500,000 participants • Supports global research on cancer, heart disease, and autoimmune diseases • Offers broad researcher access via a cloud-based platform • Identified metabolomic and genetic profiles that improve prediction of RA risk • Showed that RA patients with multiple comorbidities face higher risks of mortality and cardiovascular events, stressing the need for holistic care
	Danish Rheumatologic Biobank (DRB) [12, 96]	Blood, synovial fluid, tissue and urine	Denmark	• National Danish registry established in 2000 for RA treatment monitoring • Captures > 90% of adult RA patients on biologics in Denmark • Dual-purpose system serving as both clinical documentation tool and research registry • Monitors disease activity, medication safety, and treatment outcomes • Informs national RA treatment guidelines and quality standards • Advances personalized medicine through comprehensive longitudinal data
	Childhood Arthritis and Rheumatology Research Alliance (CARRA) [97, 98]	Plasma, serum, cells, DNA, RNA	North America	• North American pediatric rheumatology registry founded in 2002 with 10,000 + enrolled children • Collects longitudinal clinical and patient-reported data on juvenile arthritis, lupus, and other pediatric rheumatic diseases • Maintains biorepository with samples from juvenile idiopathic arthritis, SLE, dermatomyositis, scleroderma • Supports diverse research: observational studies, pharmacosurveillance, comparative effectiveness, translational science • Key findings include racial disparities in pediatric lupus outcomes and subtype-specific responses in juvenile lupus nephritis • Provides evidence for personalized treatment approaches in pediatric rheumatology

Paediatric rheumatic diseases, though rare, necessitate targeted research for advancing understanding and therapeutic development. Biospecimen collection from paediatric cohorts with rheumatic diseases presents unique ethical and logistical challenges, balancing participation rights with child protection mandates. Recent recommendations by Public and Professional Policy Committee of European Society of Human Genetics and European normative and legal framework surrounding paediatric clinical trials emphasize subsidiarity, the paediatric rule, minor protection, and burden minimization [68, 69]. Integrating minors in the consent process is crucial, ensuring their informed participation alongside legal guardian consent [70]. Future directions emphasize multi-center international paediatric biobanks, standardized practices, robust ethical frameworks, and innovative data management tools to facilitate critical research while protecting young participants [69, 70].

Among rare rheumatic diseases, IIM represent a field where international biobanking efforts are still emerging and would benefit from greater coordination and standardization. Over the past decades, numerous biobanks and registries have contributed to significant advancements in understanding and treating IIM, across both neurologic and rheumatologic disciplines [71–76]. However, many biobanks continue to operate independently, maintaining separate biospecimen collections and adhering to distinct protocols [77, 78]. These fragmented approaches highlight the ongoing need for standardized practices to maximize the impact of research in this area. Recognizing this challenges, the Myositis International Health Research Collaborative Alliance (MIHRA) has been established to foster global cooperation in myositis research.

## Opportunities and Future Perspective – the Need for Collaboration in Rare Rheumatic Diseases

The future of biobanking for rare rheumatic diseases depends on several critical advancements. The development and adoption of evidence-based guidelines for cold-chain management and sample processing protocols tailored to rare diseases are urgently needed. The current landscape remains fragmented due to differing national directives and technical methods, leading to significant heterogeneity among biobanks [99, 100].

Integrating standardized pre-analytical protocols into biobank management is also essential, especially for new facilities and high-priority specimens in established repositories. This approach is much needed as biorepositories face growing demands for quality assurance. Implementing SPREC, BRISQ or MIABIS in this context may harmonize the existing systems and align with global efforts to improve quality management [19]. Moving forward, collaboration across biobanking communities will be crucial ideally through virtual pilot projects that use shared online platforms and databases to simulate, and refine preanalytical barcode systems.

These initiatives could facilitate data sharing and remote collaborations, helping diverse biorepositories adopt consistent and interoperable practices without needing physical exchanges or on-site coordination.

Additionally, fostering international collaboration is crucial. Given the low prevalence and significant heterogeneity of rare diseases, large and diverse datasets are essential for meaningful research. Streamlined and efficient governance structures, including harmonized legal frameworks and expedited ethical review processes, are necessary to facilitate this global cooperation [101]. The Single Hub and Access point for pediatric Rheumatology in Europe (SHARE) initiative's development of the first recommendations for pediatric rheumatology collaborative research, including biobanking, exemplifies the progress that can be made through such collaboration [102].

Public funding from national and transnational entities (e.g., EU, WHO) should prioritize centralized rare disease registries, emphasizing data curation and sharing. Platforms like the Rare Disease Cures Accelerator-Data and Analytics Platform (RDCA-DAP) could offer funding towards data-enabled registries, encouraging standardized biobank linkage [103]. This approach would dismantle proprietary data silos, meet stakeholder needs, and de-risk early-stage trials, thereby incentivizing industry investment in rare disease research [104].

Academic institutions should reconsider promotion and recognition criteria by shifting away from rewarding data ownership and instead recognizing data-sharing practices and collective achievements. This shift would encourage broader participation and promote more efficient resource utilization across the rare disease research field.

**Investing in biobanking infrastructure** is another critical priority. Developing advanced IT systems for data management, secure storage facilities, and comprehensive training programs for biobank personnel will ensure the long-term sustainability and efficiency of biobanking operations [105]. Innovative technologies, such as permissioned blockchain frameworks, can enhance transparency and traceability in biobanking processes [106]. Additionally, tools like text mining can facilitate easier data extraction from electronic medical records, improving data collection efficiency [107].

While innovative technologies can enhance data extraction efficiency, they may be difficult to be implemented in resource-restricted environments. In these settings, it is crucial to prioritize foundational infrastructure, such as basic cold chain solutions and essential data management systems, to ensure at least minimal compliance with biobanking standards. Focusing on biospecimens that are easier to handle, like DNA and formalin-fixed paraffin embedded tissues, may be more feasible. In contrast, specimens such as RNA, proteins, or fresh tissues, which are used for metabolomics and proteomics, require strict handling and cold chain control, making them impractical where processing protocols are hard to maintain [108].

**A move towards integrative, open, and community-driven registries** is also necessary to overcome the limitations of existing systems. Such registries should align and harmonize conflicting standards, make data and code publicly available, and engage community members in governance, thereby promoting sustainability and longevity [109].

Tailoring biobank operations to the specific types of samples and assays needed in each context is essential for maintaining high-quality research outcomes. Furthermore, securing funding—whether through grants, international collaborations, or partnerships—can provide the necessary resources to build this foundational infrastructure and gradually scale up operations.

Engaging stakeholders, including patients and patient organizations, in governance processes will further build trust and ensure that biobanking initiatives reflect diverse perspectives. Moreover, promoting patient recruitment, especially in specific groups such as adolescents, requires creative recruitment and scheduling strategies tailored to the needs of these populations [110].

**Educational initiatives** aimed at young translational scientists are vital for accelerating advancements in the field. Encouraging researchers to think beyond their institutions and embrace collaborative approaches early in their careers can foster more innovative and inclusive research efforts [111, 112].

Organizations such as NCATS (National Center for Advancing Translational Sciences), RDCRN (Rare Disease Clinical Research Network) and MIHRA have established teams working in this space [113, 114]. Led by an interdisciplinary panel, MIHRA group is developing an evidence-based protocol for biospecimen processing and storage. This incorporates qualitative interviews to identify barriers in the field based on existing evidence of feasibility and logistics, while emphasizing biospecimen validity.

## Conclusions

Biobanking has revolutionized rare disease research by enabling breakthrough discoveries and fostering national and international collaborations. As the field progresses towards personalized medicine, biobanks will become increasingly indispensable in advancing diagnostics, therapeutics, and biomarker discovery. Despite their transformative impact, several unmet challenges persist. Ethical, legal and social concerns regarding data privacy patient confidentiality, and equitable access must be continually addressed through robust governance frameworks and ethical oversight. Additionally, public awareness, building trust among the donors, transparency, standardization and harmonization, sustainability and funding and integration with digital health technologies will be essential for ensuring the long-term efficiency and interoperability of biorepositories. These challenges can be met by engaging multi-stakeholders, enhanced public participation, cross-disciplinary collaboration, supportive policymaking and better integration with digital health tools. By strengthening these areas, biobanking will continue to drive innovation and accelerate rare disease research.

## Box. Key Initiatives in Biospecimen and Data Standardization

**Table:** 

Initiative	Full Name	Main Purpose	Application Area	Relevance
SPREC [32]	Standard PREanalytical Code	Provides a standardized coding system for preanalytical variables during specimen collection, processing, and storage	Biobanking	Enhances reproducibility by documenting factors affecting biospecimen integrity; adaptable across clinical and non-clinical settings
BRISQ [31]	Biospecimen Reporting for Improved Study Quality	Offers a tiered reporting system to improve consistency and transparency in human biospecimen research	Reporting Standards	Improves reliability and comparability of study results; developed through multi-organization consensus including NCI and ISBER
MIABIS [33]	Minimum Information About Biobank Data Sharing	Standardizes core metadata required for biobank interoperability and data sharing	Biobank Metadata	Facilitates collaboration by harmonizing biorepository data structures; supports resource discoverability
FAIR [49]	Findable, Accessible, Interoperable, Reusable	Establishes principles to improve data sharing, reuse, and machine-readability in research	Data Stewardship	Widely adopted for health data; enables integration and reuse across systems; supported by initiatives like ELIXIR and GO FAIR
CoBRA [46]	Citation of BioResources in Research Articles	Recommends standardized citation of bioresources in scientific publications	Reporting/Citation	Enhances traceability, credit, and reuse of bioresources; encourages transparency and resource visibility

### Key References

- Snapes E, Astrin JJ, Bertheussen Krüger N, Grossman GH, Hendrickson E, Miller N, et al. Updating International Society for Biological and Environmental Repositories Best Practices, Fifth Edition: A New Process for Relevance in an Evolving Landscape. Biopreservation and Biobanking. 2023 Dec;21(6):537–46.

- *Addresses the evolving challenges in biobaking and repository management.*

- Kirwan JA, Brennan L, Broadhurst D, Fiehn O, Cascante M, Dunn WB, et al. Preanalytical Processing and Biobanking Procedures of Biological Samples for Metabolomics Research: A White Paper, Community Perspective (for “Precision Medicine and Pharmacometabolomics Task Group”-The Metabolomics Society Initiative). Clin Chem. 2018 Aug;64(8):1158–82.

- *Addresses and emphasizes on the importance of standardized preanalytical and biobanking procedures in metabolomics research. The authors emphasize that variations in sample collection, processing, and storage can significantly impact the metabolomic profiles obtained, potentially leading to inconsistent or misleading results.*

- Howard HC, Mascalzoni D, Mabile L, Houeland G, Rial-Sebbag E, Cambon-Thomsen A. How to responsibly acknowledge research work in the era of big data and biobanks: ethical aspects of the Bioresource Research Impact Factor (BRIF). J Community Genet. 2018 Apr;9(2):169–76.

- *Addresses an important topic: the challenges of recognizing and crediting the contributions of researchers and institutions that develop and maintain bioresources, such as biobanks.*

- Rush A, Byrne JA, Watson PH. Applying Findable, Accessible, Interoperable, and Reusable Principles to Biospecimens and Biobanks. Biopreserv Biobank. 2024 Dec;22(6):550–6.

- *Emphasizes that applying FAIR principles to biospecimens and biobanks can improve data sharing, enhance research productivity, and ensure efficient use of resources in health research.*

- Beyer C, Distler JHW, Allanore Y, Aringer M, Avouac J, Czirják L, et al. EUSTAR biobanking: recommendations for the collection, storage and distribution of biospecimens in scleroderma research. Ann Rheum Dis. 2011 Jul;70(7):1178–82.

- *Serves as a foundational resource for researchers involved in Systemic sclerosis studies, providing comprehensive guidelines to standardize biobanking practices and enhance the quality of systemic sclerosis research.*

- Rider LG, Dankó K, Miller FW. Myositis registries and biorepositories: powerful tools to advance clinical, epidemiologic and pathogenic research. Curr Opin Rheumatol. 2014 Nov;26(6):724–41.

- *Highlights the significant role of clinical registries and biorepositories in enhancing our understanding of idiopathic inflammatory myopathies. Provides a comprehensive overview of the existing registries and biobanks in idiopathic inflammatory myopathies.*

## Data Availability

No datasets were generated or analysed during the current study.
